# Supplementary material for: Printing two-dimensional gallium phosphate out of liquid metal
Source: Nat Commun. 2018 Sep 6;9:3618. doi: 10.1038/s41467-018-06124-1 (PMC6127148; doi:10.1038/s41467-018-06124-1)
Supplement: Supplementary file 1 — Supplementary Information [file 41467_2018_6124_MOESM1_ESM.pdf]

# Supplementary Information

## Printing two-dimensional gallium phosphate out of liquid metal

**Syed et al.**

## Supplementary Figures

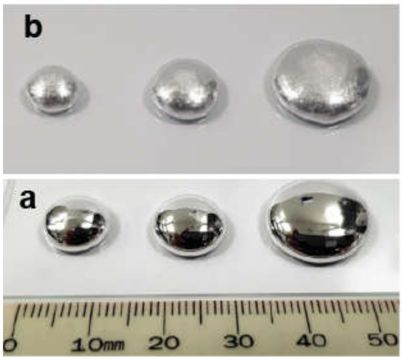


**Supplementary Figure 1 . a** Fresh liquid gallium droplets with different diameters before the formation of $Ga_2O_3$ oxide skin. **b** Gallium droplets with surface oxide skin.

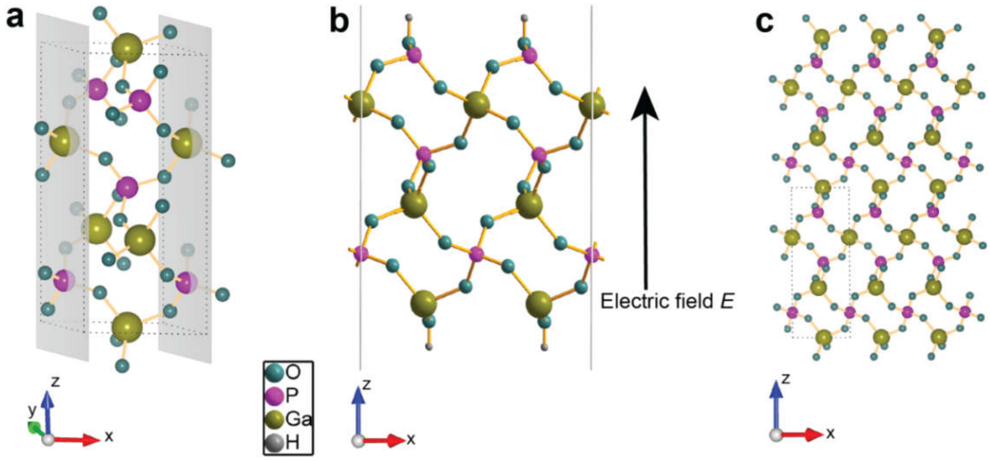


**Supplementary Figure 2. a** 1 unit cell of $GaPO_4$ along z axis. **b** A periodic 2×1 unit cell of $GaPO_4$ with a (100) plane cut from the trigonal bulk phase. This show the top and bottom oxygen atoms in the slab are hydrogen terminated. **c**. Crystal structure of few unit cell thick $GaPO_4$ along z axis, showing the non-centrosymmetric structure is present for mono to several-unit cell thick $GaPO_4$.

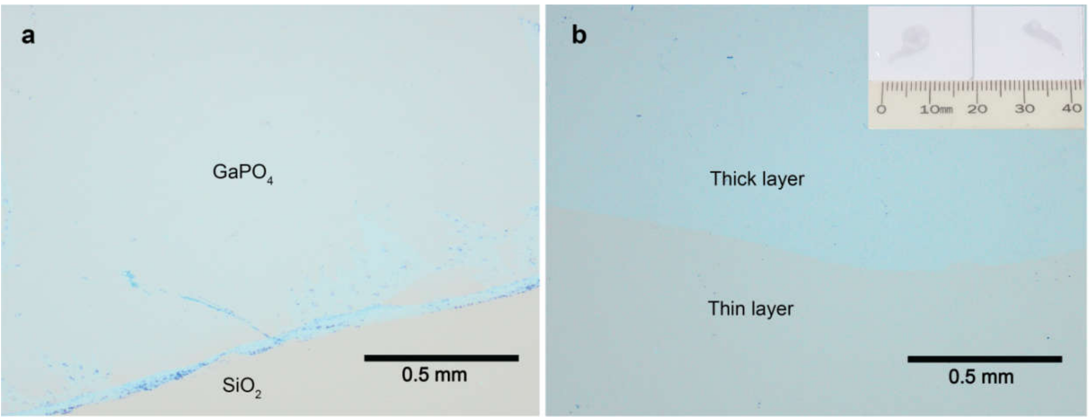


**Supplementary Figure 3. a-b** Optical images of synthesised $GaPO_4$ showing homogeneous nanosheets featuring lateral dimensions spaning several millimetres. The inset of Fig. b is showing centimetre sized $Ga_2O_3$ nanosheets deposited onto glass.

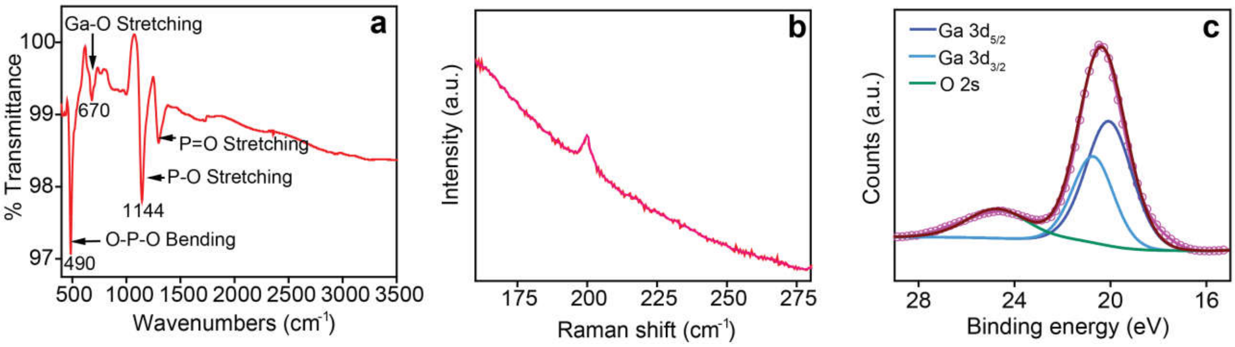


**Supplementary Figure 4. a** FTIR of the synthesised $GaPO_4$ nanosheets. The spectrum is characterised by strong absorptions features between 1250 and 1100 $cm^{-1}$ which can be attributed to P-O stretching. The peak located near 500 $cm^{-1}$ is due to O-P-O bending and the peak within the region of 700 to 600 $cm^{-1}$ is assumed to be generated by Ga-O stretching modes. All of these peaks match well with the previous reports[1]. **b** Raman spectra of the synthesised 2D $Ga_2O_3$ nano sheet featuring characteristic peak at ~199.8 $cm^{-1}$.**c** XPS results of Ga 3*d* region of $Ga_2O_3$ flakes. All the XPS spectra are obtained by calibrating C1s peak

located at 284.80 eV[2]. The Raman spectra and XPS analysis of the resulting oxide layer is in good agreement with literature reports for the $Ga_2O_3$[3,4].

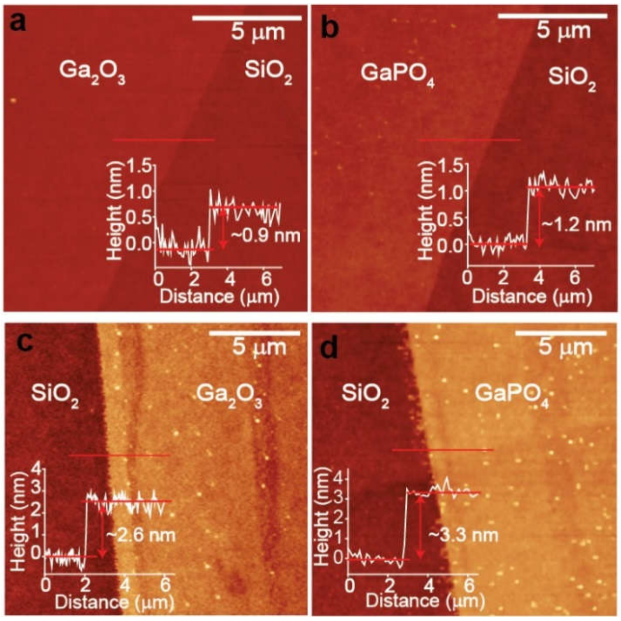


**Supplementary Figure 5.** AFM image of 2D $Ga_2O_3$ sheets imaged before **(a, c)** and after **(b, d)** conversion into 2D $GaPO_4$.

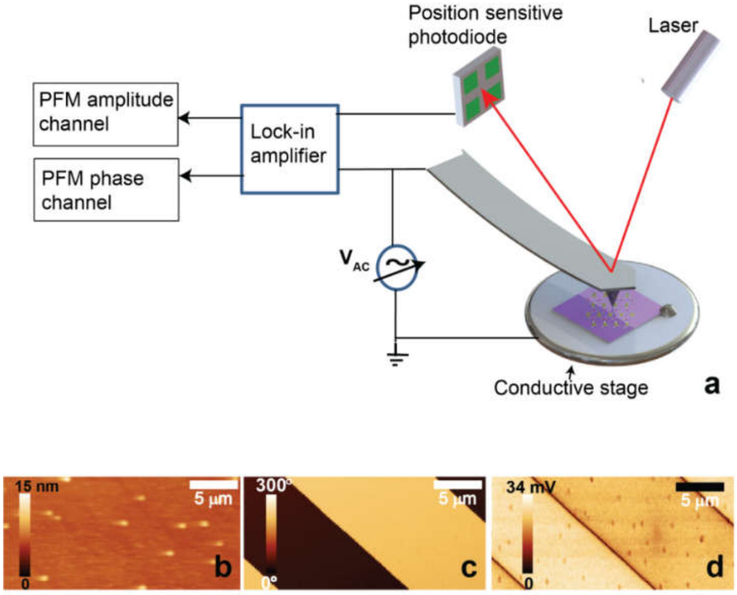


**Supplementary Figure 6. a** Schematic illustration showing the PFM measurement process.

**b** Topography of a PPLN substrate, **c** PFM phase, and; **d** PFM amplitude of a standard PPLN sample as reference with a PR drive amplitude of 10 V at 17 kHz.

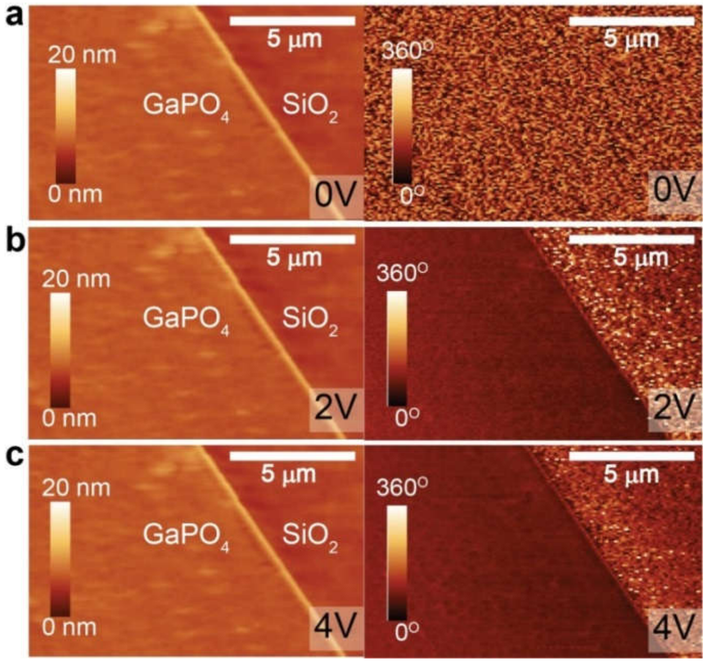


**Supplementary Figure 7. a-c** Topography and phase images at different AC voltages during PFM measurements for the $GaPO_4$ flake with unit cell thickness. The images show that the morphology of the $GaPO_4$ flake is not affected while applying high electric field on its surface. The phase images reveal significant phase variations between the $GaPO_4$ film and substrate.

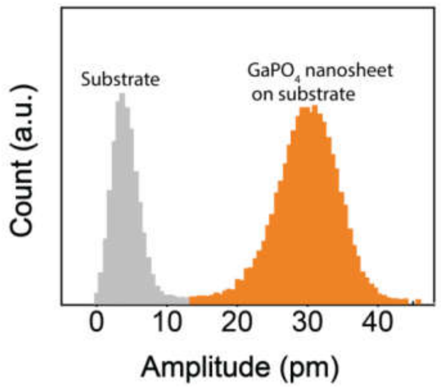


**Supplementary Figure 8.** Statistical distribution of the piezoresponse amplitude between the $GaPO_4$ nanosheet and the substrate.

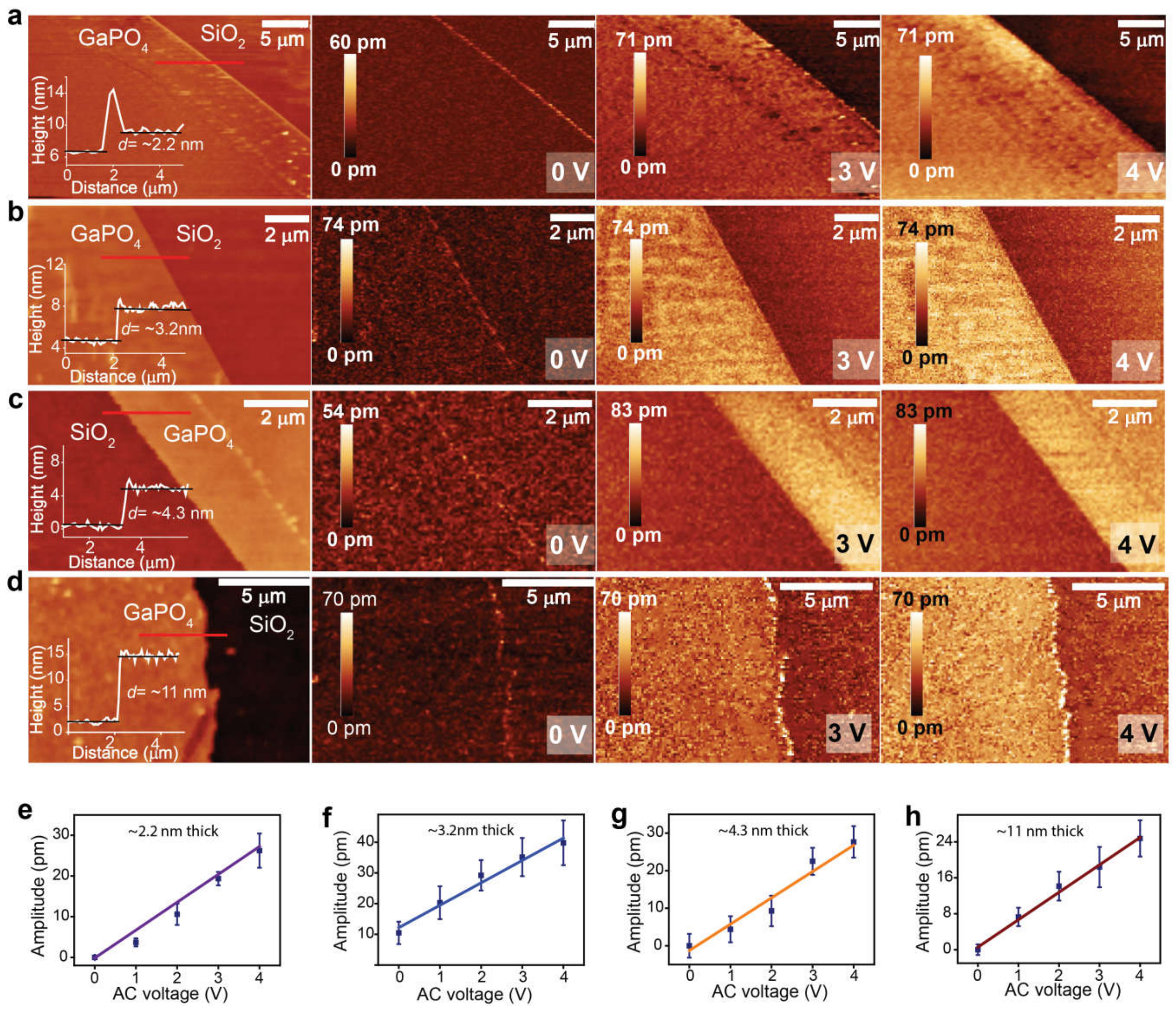


**Supplementary Figure 9. a-d** Topography and vertical piezoresponse amplitude profiles for

different 2D $GaPO_4$ nanosheets at various AC driving voltages. **e-h** Average piezoresponse amplitude as a function of the applied AC voltage for different $GaPO_4$ nanosheets obtained from the statistical distributions. Error bars denote the standard deviations.

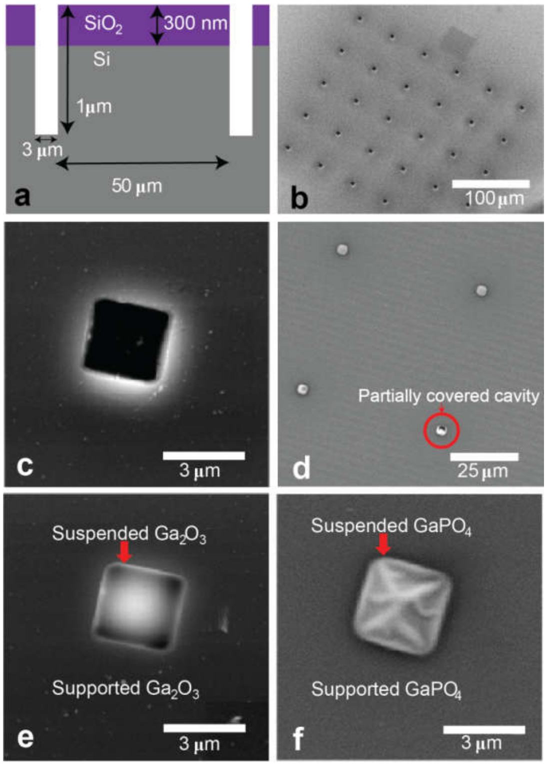


**Supplementary Figure 10. a** The schematic for the cross section of the $SiO_2$/Si wafer with micro cavities. SEM images of **b** array of micro cavities, **c** an uncovered square hole, **d** fully and partially covered cavities by $GaPO_4$ nanosheet , **e** cavity covered with $Ga_2O_3$ flake before phosphatisation process which is showing an even bulged surface area, **f** hole covered with $GaPO_4$ nanosheet showing a wrinkled surface.

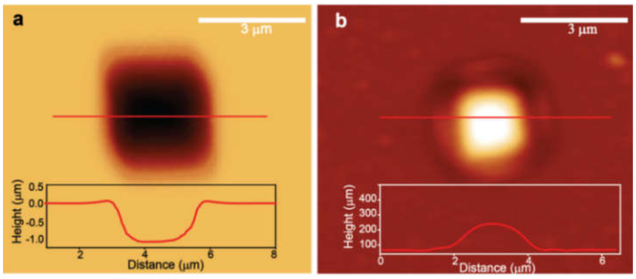


**Supplementary Figure 11. a** AFM topography of an uncovered square cavity and its depth profile along the red line. **b** AFM image of a cavity covered with $GaPO_4$ nanosheet and its height profile along the red line.

r

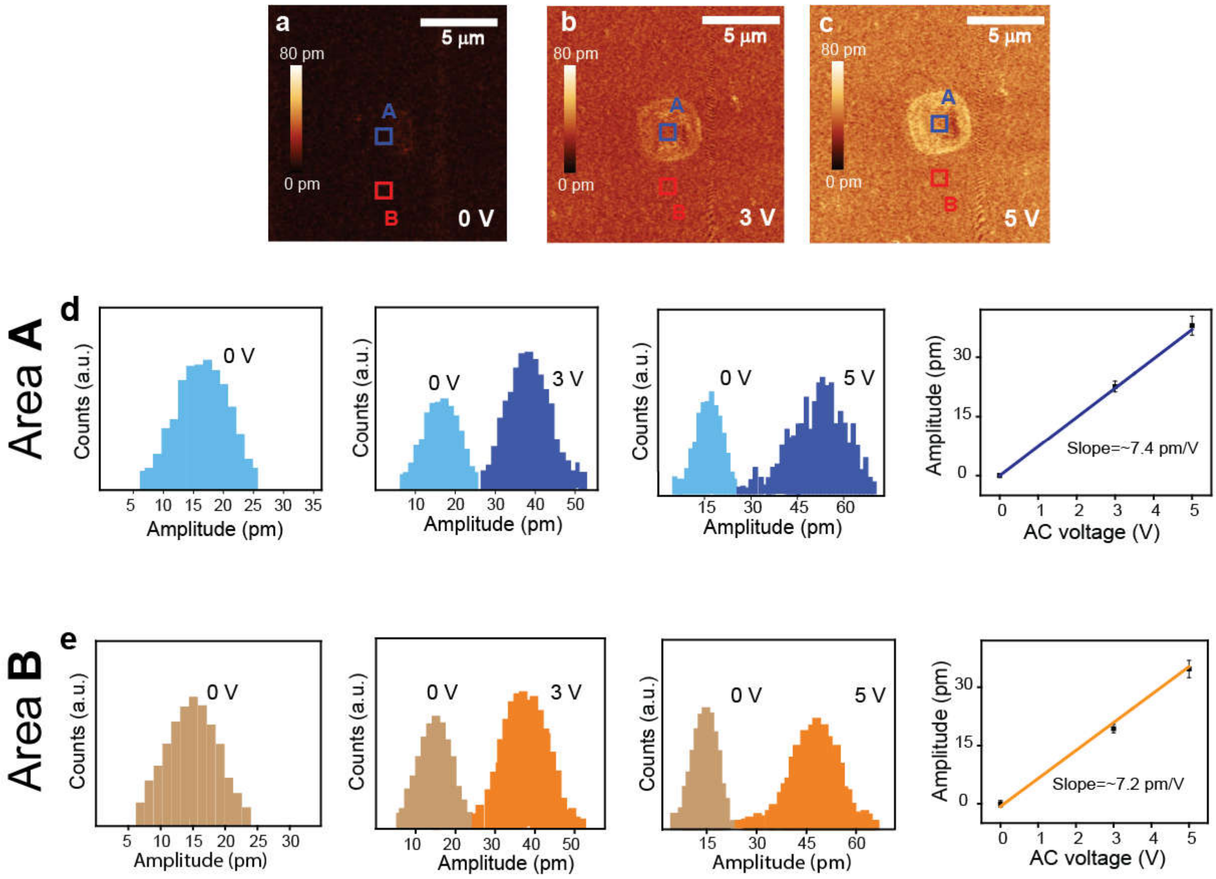


**Supplementary Figure 12.** Vertical piezoresponse of free-standing $GaPO_4$ nanosheet at **(a)** 0V (background) and **(b)** 3V, **(c)** 5V. **d-e** Statistical distributions of the piezoresponse amplitude variation for the flat and uniform surface area of free standing (area A) $GaPO_4$ and supported (area B) $GaPO_4$ films at different voltages (3V and 5V) and the background (0V).

The slope of the piezoresponse amplitude *vs* AC voltage graphs in both cases are in good agreement.

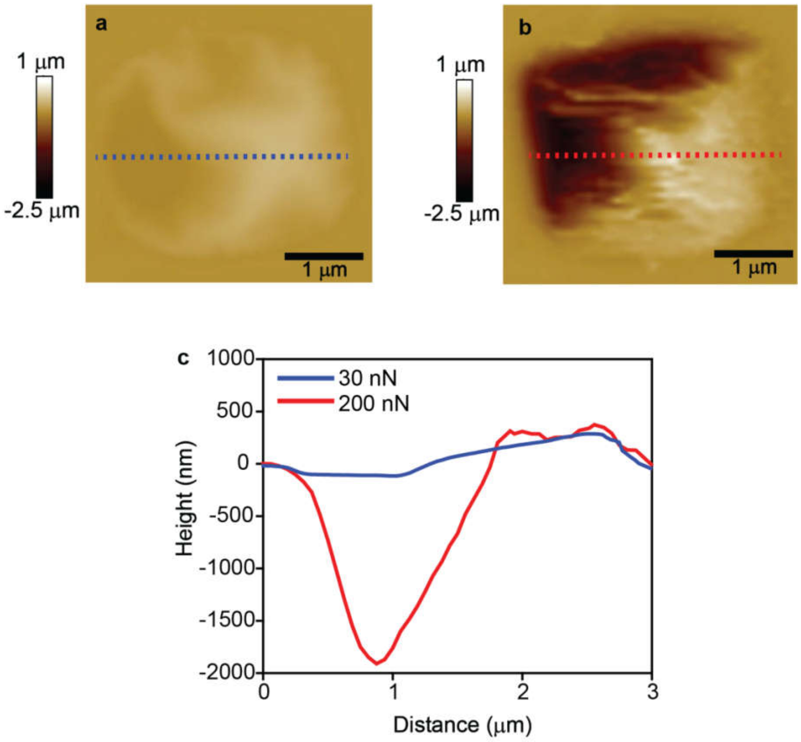


**Supplementary Figure 13. a** AFM image of a suspended $GaPO_4$ nanosheet (before the breakdown occurred) on a cavity with 2 μm depth. **b** AFM image of the fractured membrane after applying 200 nN load to the tip. **c** Cross sections of the AFM topographic maps of the free standing $GaPO_4$ at two different forces.

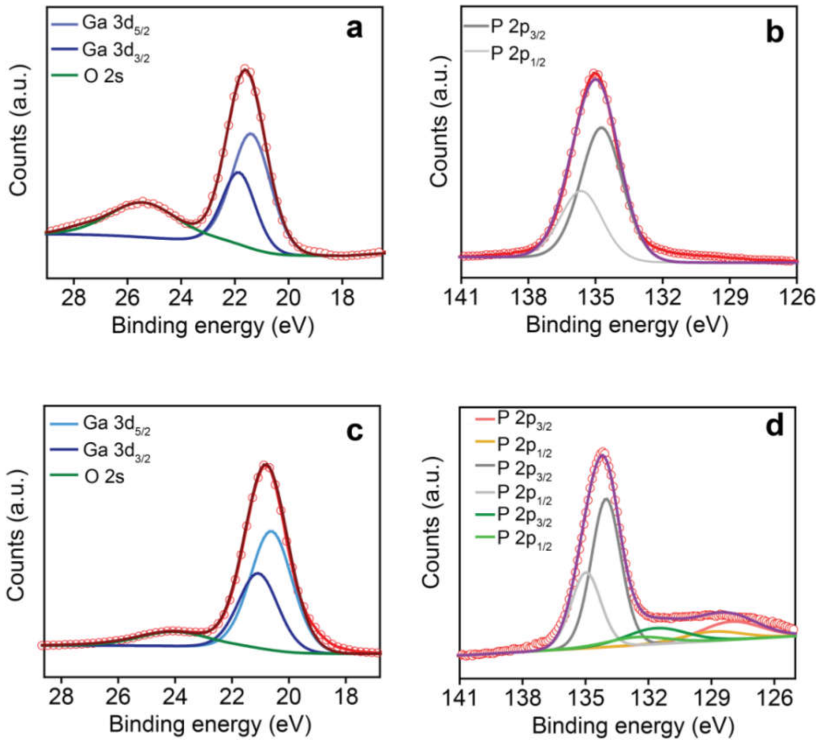


**Supplementary Figure 14.** XPS results of **a** Ga 3*d* and **b** phosphorus 2*p* regions of the synthesised $GaPO_4$ annealed at 600 °C. XPS results of **c** Ga 3*d* and **d** phosphorus 2*p* regions of the synthesised $GaPO_4$ annealed at 700 °C.

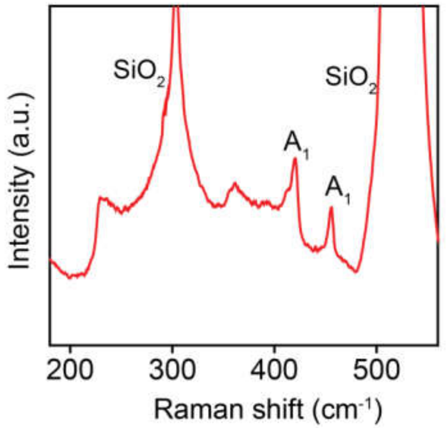


**Supplementary Figure 15.** Raman spectrum of $GaPO_4$ nanosheet on $SiO_2$ substrate annealed at 600 °C.

## Supplementary Tables

**Supplementary Table 1.** Comparison of $d_{33}$ between some previously reported 2D films and this work.

| Material | Thickness (nm) | Theoretical simulation $d_{33}$(pm $V^{-1}$) | Experimental effective $d_{33}$ (pm $V^{-1}$) | Temperature stability (°C) | Ref. |
|---|---|---|---|---|---|
| ZnO | ~2 | - | 23.7 | < 350 | [5] |
| CdS | 2-3 | - | 16.4[a] | < 600 | [6] |
| Graphene on $SiO_2$ | 0.34 | 1,400[b] | | - | [7] |
| MoSSe | 1.4 | 5.24 | - | < 550 | [8] |
| MoSeTe | 1.4 | 6.21 | - | < 550 | [8] |
| MoSTe | 1.4 | 10.57 | - | < 550 | [8] |
| WSSe | 1.4 | 5.31 | - | < 550 | [8] |
| WSeTe | 1.4 | 6.71 | - | < 550 | [8] |
| WSTe | 1.4 | 9.27 | - | < 550 | [8] |
| $C_3N_4$ | ~2 | 0 | 1 | - | [9] |
| $MoS_2$ | 0.7 | - | 1.35±0.24 (flexoelectric effect) | < 550 | [10] |
| α-$GaPO_4$ | ~1.1 | ~8.5 | 7.5±0.8 | < 700 | This work |

[a] The effective $d_{33}$ was later multiplied by 2, resulting in a piezoelectric constant of 32.4 pm $V^{-1}$.

[b] The number is extremely high and has not been confirmed by any other research groups. The value is over 5 times the thickness of the graphene unit cell. So such a measurement is practically unlikely.

**Supplementary Table 2.** Comparison of $d_{33}$ between some previously reported bulk materials and this work.

| Material | Thickness (nm) | Theoretical simulation $d_{33}$(pm $V^{-1}$) | Experimental effective $d_{33}$ (pm $V^{-1}$) | Ref. |
|---|---|---|---|---|
| GaN | ~140 | - | 3.1 | [11,12] |
| AlN | ~450 | - | 5.1 | [11,12] |
| ZnO nanobelts | ~65 | - | 14-26 | [13] |
| ultra-thin α-$GaPO_4$ film | 1.1 | ~8.5 | 7.5±0.8 | This work |

## Supplementary Notes

## Supplementary Note 1: Synthesis of large $Ga_2O_3$ nanosheets

The naturally occurring 2D layer of $Ga_2O_3$ fully covers the surface of the droplet in oxygen containing environment. The oxide layer is transferred by firmly touching the target substrate against the gallium droplet as reported to our previous published work[14]. Different gallium droplets with varying diameters of 4 to 20 mm were chosen (Supplementary Fig. 1a-b) to harvest $Ga_2O_3$ nanosheets. Based on the technique used for transferring the oxide layer, the size of the gallium droplet is not a limiting factor. The $Ga_2O_3$ sheets with lateral dimension of several millimetres were obtained from the gallium droplets with diameter range 1-2 centimetre. These large $Ga_2O_3$ sheets were later transformed into $GaPO_4$ by using a chemical vapour method.

However, the experimental limitations for fabricating larger nanosheets mainly depend on the applied force and the approaching angle of the target substrate that is brought into contact with the liquid metal. If the larger liquid-metal droplet is not uniformly touched by the substrate, the transferred area of $Ga_2O_3$ sheets may lack homogeneity. Conversely, when excessive force is used on the droplet the probability of metal inclusions increases during the oxide layer transfer occurs. The metal inclusions can be cleaned and removed by following the methods proposed by Jing Liu *et al*[15]. During the separation of the 2D nanosheets from the gallium metal, the possibility of overlapping and folding of the monolayer along the edges result in increased flake thickness, which can also be considered as one of the limitations to this process (Supplementary Fig. 3a).

## Supplementary Note 2: Synthesis of $GaPO_4$ nanosheets of differing thicknesses

2D $Ga_2O_3$ sheets were produced using the van der Waals printing process on a liquid gallium droplet. The isolated oxide sheets were transformed to 2D $GaPO_4$ using a chemical vapour method. The interfacial oxide of the gallium metal has been shown to grow in a self-limiting reaction[16] and the thickness of the transferred $Ga_2O_3$ nanosheets is defined by the Cabrera-Mott process[17,18]. The thickness of the $Ga_2O_3$ sheets obtained through exfoliation was found to be approximately 0.8-1 nm. Occasionally different thicknesses reaching 2-3.5 nm were observed, primarily around the edges. These thicker areas arise during the separation technique of 2D $Ga_2O_3$ sheets from the gallium metal due to folding of the monolayer sheets.

A strategy towards the targeted synthesis of thicker $Ga_2O_3$ sheets can be obtained by multiple subsequent van der Waals exfoliation/printing processes onto the same substrate. The thickness of the obtained 2D $GaPO_4$ was found to mainly depend on the original thickness of the exfoliated 2D $Ga_2O_3$ sheets. The thickness profiles of two different $Ga_2O_3$ oxide nanosheets (0.9 and 2.6 nm thick) are presented in Supplementary Fig. 5 a,c, before the phosphatisation process. The thickness of the transformed 2D $GaPO_4$ was consistently 1.3 times larger than that of the original $Ga_2O_3$ nanosheets (Supplementary Fig. 5b,d). The observed change in thickness arises due to the recrystallisation process and the incorporation of phosphate ions into the material.

## Supplementary Note 3: Thermal stability of the synthesized 2D $GaPO_4$

The thermal stability of the synthesised $GaPO_4$ nanosheets was investigated *via* XPS of annealed samples. For samples annealed at 600 °C, the characteristic main broad peak for the Ga 3*d* region is centered at 21.4 eV and the main broad peak in the phosphorus 2*p* region

is centered at a binding energy of 134.7 eV (Supplementary Fig 14a-b), which is consistent with unannealed $GaPO_4$ (Fig. 3c), evidencing thermal stability at this temperature. When annealed at 700 °C (Supplementary Fig 14c-d), the XPS results show a significant shift of the Ga 3d peaks to a lower binding energy (20.6 eV) which resembles the spectrum of $Ga_2O_3$[4]. Furthermore, several new P 2*p* peaks appear at lower binding energies ranging from 126 to 132 eV, indicating the partial transformation from phosphate to other phosphorous componds[19,20].

Raman spectrum of $GaPO_4$ nanosheet on $SiO_2$ substrate annealed at 600 °C shows two strong Raman peaks at ~420.8 and ~456.3 $cm^{-1}$ (Supplementary Fig 15) that are in good agreement with the peak positions of the original $GaPO_4$ nanosheet ( Fig. 3b), validating the stability of the 2D $GaPO_4$ at temperatures up to 600 °C.

## Supplementary References


1 Hirano, S. & Kim, P. Growth of gallium orthophosphate single crystals in acidic hydrothermal solutions. *J. Mater. Sci.* **26**, 2805-2808 (1991).

2 Mohanty, N., Moore, D. & Xu, Z. Nanotomy-based production of transferable and dispersible graphene nanostructures of controlled shape and size. *Nat. Commun.* **3**, 844 (2012).

3 Kranert, C., Sturm, C., Schmidt-Grund, R. & Grundmann, M. Raman tensor elements of β-$Ga_2O_3$. *Sci. Rep.* **6**, 35964 (2016).

4 Zhang, W. *et al.* Liquid metal/metal oxide frameworks. *Adv. Funct. Mater.* **24**, 3799-3807 (2014).

5 Wang, L. *et al.* Ultrathin piezotronic transistors with 2 nm channel lengths. *ACS Nano* **12**, 4903–4908 (2018).

6 Wang, X. *et al.* Subatomic deformation driven by vertical piezoelectricity from CdS ultrathin films. *Sci. Adv.* **2**, e1600209 (2016).

7 Da Cunha Rodrigues, G. *et al.* Strong piezoelectricity in single-layer graphene deposited on $SiO_2$ grating substrates. *Nat. Commun.* **6**, 7572 (2015).

8 Dong, L., Lou, J. & Shenoy, V. B. Large in-plane and vertical piezoelectricity in janus transition metal dichalchogenides. *ACS Nano* **11**, 8242-8248 (2017).

9 Zelisko, M. *et al.* Anomalous piezoelectricity in two-dimensional graphene nitride nanosheets. *Nat. Commun.* **5**, 4284 (2014).

10 Brennan, C. J. *et al.* Out-of-plane electromechanical response of monolayer molybdenum disulfide measured by piezoresponse force microscopy. *Nano Lett.* **17**, 5464-5471 (2017).

11 Duerloo, K.-A. N., Ong, M. T. & Reed, E. J. Intrinsic piezoelectricity in two-dimensional materials. *J. Phys. Chem. Lett.* **3**, 2871-2876 (2012).

12 Lueng, C., Chan, H. L., Surya, C. & Choy, C. Piezoelectric coefficient of aluminum nitride and gallium nitride. *J. Appl. Phys.* **88**, 5360-5363 (2000).

13 Zhao, M.-H., Wang, Z.-L. & Mao, S. X. Piezoelectric characterization of individual zinc oxide nanobelt probed by piezoresponse force microscope. *Nano Lett.* **4**, 587-590 (2004).

14 Zavabeti, A. *et al.* A liquid metal reaction environment for the room-temperature synthesis of atomically thin metal oxides. *Science* **358**, 332-335 (2017).

15 Ma, R., Zhou, Y. & Liu, J. Erasing and Correction of Liquid Metal Printed Electronics Made of Gallium Alloy Ink from the Substrate. *arXiv:1706.01457* (2017).

16 Carey, B. J. *et al.* Wafer-scale two-dimensional semiconductors from printed oxide skin of liquid metals. *Nat. Commun.* **8**, 14482 (2017).

17 Cabrera, N. & Mott, N. F. Theory of the oxidation of metals. *Rep. Prog. Phys.* **12**, 163-184 (1949).

18 Daeneke, T. *et al.* Liquid metals: fundamentals and applications in chemistry. *Chem. Soc. Rev.* **47**, 4073-4111 (2018).

19 Son, C. Y., Kwak, I. H., Lim, Y. R. & Park, J. FeP and $FeP_2$ nanowires for efficient electrocatalytic hydrogen evolution reaction. *Chem. Comm.* **52**, 2819-2822 (2016).

20 Huang, S. R., Lu, X., Wang, X., Barnett, A. M. & Opila, R. L. in *Photovoltaic Specialists Conference.* 1-4 (IEEE).
